# Donning and Doffing of Personal Protective Equipment: Perceived Effectiveness of Virtual Simulation Training to Decrease COVID-19 Transmission and Contraction

**DOI:** 10.7759/cureus.22943

**Published:** 2022-03-07

**Authors:** Cynthia Mosher, Fareeda Mukhtar, Nuha Alnaami, Yara A Akkielah, Joud Alsharif, Tariq Khan, Huseyin Cahit Taskiran, Muhammad Zafar

**Affiliations:** 1 Department of Clinical Skills, Alfaisal University College of Medicine, Riyadh, SAU

**Keywords:** virtual, distance, simulation, personal protective equipment, doffing, donning, covid-19, ppe

## Abstract

Introduction

The COVID-19 pandemic exposed gaps in the knowledge of correct donning and doffing of personal protective equipment (PPE) among healthcare workers, causing hospitals to ramp up training. However, social distancing measures forced most institutions and workplaces to shift to remote operations, allowing only essential personnel onsite. Virtual simulation is a growing trend in healthcare simulation education, even more so in this pandemic era. Yet, we have found no evidence of the perceived effectiveness of virtual simulation for training healthcare providers in the proper donning and doffing of PPE. This study aims to determine learner perceptions of the effectiveness of a virtual simulation PPE training module.

Methods

To address this gap, we used a virtual simulation training module in an online format to determine the perceived efficacy of this method of instruction with the contribution of a variety of healthcare providers and trainees, including physicians, surgeons, pharmacists, dentists, and nurses.

Results

We found a statistically significant difference in the confidence level of observing best practices of donning and doffing PPE before and after the training sessions. We also found that participants believe virtual simulation can be an effective educational tool for clinical skills.

Conclusions

This paper presents an international, guideline-based virtual simulation training module that can serve to educate, train, and assess healthcare workers in the proper sequence and technique of donning (putting on), doffing (removing), and disposing of PPE without contaminating themselves or others.

## Introduction

As millions of people around the world are staying home and socially distancing to minimize the transmission of severe acute respiratory syndrome coronavirus 2 (SARS-CoV-2), the global healthcare workforce continues to wake up every day to face it head-on. The burden of illness continues to exceed healthcare capacity in many areas of the world, and the risk of infection of healthcare providers is of constant concern with reports of up to 20% infection rate. As the pandemic continues, all healthcare providers must wear personal protective equipment (PPE), not just those in direct contact with infected patients. Ensuring the provision of PPE is the most tangible organizational measure. However, COVID-19 has exposed potential gaps in the knowledge and skills of correct PPE use, causing healthcare institutions to ramp up staff training.

With social distancing measures in practice, remote operations are now common for most educational institutions and workplaces. As such, we believe that an online module based on international guidelines regarding the proper usage and disposal of PPE is a necessity. The purpose of this investigation is to assess the perceived effectiveness of online simulation training in the proper donning and doffing of PPE. While this study is not designed to determine the ability of virtual simulation training to prevent contraction and spread of infection, we believe it can lead to increased knowledge and awareness of correct donning and doffing of PPE, which is expected to contribute to a decrease in the transmission and contraction of SARS-CoV-2 and other highly infectious agents. For this study, we use the term "virtual" to include distance, remote, and online.

Among the victims of the COVID-19 pandemic are some of the most important people serving the frontlines - our physicians, physicians in training, nurses, and other healthcare workers who face the disease head-on every day. China reported that more than 3,300 healthcare workers were infected with SARS-CoV-2, with nearly 22 deaths recorded and widespread concomitant infection of family members [1]. Italy and the United States reported a nearly 20% infection rate of healthcare providers with severe outcomes and death reported broadly [2,3]. With such concerning infection rates, it is critical to ensure the safety of healthcare workers. The Centers for Disease Control cited that training on preventive measures, including hand hygiene and PPE use, is an important factor in safeguarding against transmission in healthcare settings [3].

During a pandemic, the proper use of PPE is essential; however, its effectiveness in protecting from infection is highly dependent on the user. Studies conducted in the United Kingdom and the United States showed healthcare workers were frequently contaminated because of post-doffing errors [4,5]. To be effective, all PPE items (mask, gown, eye-protective equipment, and gloves) should be worn together. Additionally, effective training is essential for correct donning, doffing, and disposal of PPE equipment [6]. Studies in Italy found that due to long working hours and working above the usual capacity, the correct safety procedures for PPE (e.g., donning and doffing, fit test) are not being followed closely [7].

In a recent observational study, 90% of staff did not use the correct doffing technique, and 14% of physicians reported never receiving PPE training [6]. Ippolito et al. surveyed a broad population of healthcare workers in Italy during the pandemic, wherein 65% of respondents said that they had not received on-the-job protection training related to the medical care of patients infected with the coronavirus and had not been trained in the use and disposal of PPE [7]. John et al. [8] found suboptimal education in the correct use of PPE to be a major contributing factor to suboptimal practices, while Abualenain et al. [9], in their pre-intervention needs assessment drill, found the average score of healthcare providers for donning and doffing items was a mere 37%.

Simulation training in the donning and doffing of PPE is effective in enhancing provider safety and promoting buy-in to the meticulous practice of proper technique [5,10,11]. It is important to note that medical simulation is considered one of the most dynamically developing fields of medical education. It prepares medical personnel to work with patients, allowing them to make mistakes, draw conclusions, and learn without compromising patient safety. Many studies have proven that training via simulation is effective [12]. The effectiveness of online simulation training has also been studied and shown to significantly increase the learner’s knowledge and attitude [13]. Additionally, PPE simulation training for medical students proved to significantly reduce the self-contamination rate while doffing and improved their confidence in their ability to properly doff without supervision [14].

In the face of the COVID-19 pandemic, PPE training and practice are a major concern. COVID-19 has shone a spotlight on simulation as an adaptive tool as it offers a robust and comprehensive strategy that is welcome in these uncertain times [12]. With government-mandated social distancing and shifting education online, simulation training in donning and doffing of PPE and studying its effectiveness are of prime importance. We have the opportunity to take the proven value of simulation training into the realm of online learning to provide an effective alternative for training large numbers of individuals in implementing best practices and offer a major contribution to the field of education, awareness, and training to help combat the spread of COVID-19 and other infectious diseases.

Simulation in healthcare provides us with limitless, flexible, and widespread options for education and training and will continue to be a vital cog in the wheels of effective healthcare practices as it is a tool for protocol development and refinement, an educational platform, a tool to uncover safety gaps, a training technique for healthcare workers in unfamiliar roles, and a catalyst for team-based training. Much like crash testing a car, simulation allows those in charge to observe, reflect, and refine the proposed protocols without risking harm to the healthcare workers or their patients. This process is important to ensure a consistent approach and anticipate potential problems down the line before real-time implementation [15].

This article was previously presented as a poster in Alfaisal University’s 12th Annual Research Day and the Advancing Healthcare Innovation Summit 2021.

## Materials and methods

We addressed the core research aims of this study using a pre- and post-survey. We intended to probe healthcare worker perceptions regarding the effectiveness of virtual simulation training in PPE donning and doffing. We observed the Key Elements to Report for Simulation-Based Research [16] in our methods, aiming for quality in our reporting.

Design and setting

This cross-sectional study used a pre- and post-simulation training survey as well as a virtual microsimulation training module. After a thorough literature review in the field, the surveys were designed using Bloom’s taxonomy domains of learning: affective, cognitive, and psychomotor [17]. They measured eight constructs of perceptions of knowledge and application regarding PPE donning and doffing and were rated on a five-point Likert scale (ranging from 1 = strongly disagree to 5 = strongly agree). Appendix I lists the questions in both surveys. After completing the pre-simulation survey, participants completed the online microsimulation training module in donning and doffing of PPE. Thereafter, they completed the post-simulation survey. Both the survey completion and simulation training modules were conducted online from participants’ locations using their own devices and internet access.

Sample

Given the originality of our survey, the 10:1 rule [18-21] was used to calculate the minimum sample size (10 participants per survey item, n = 90). No prior effect size or power analysis was done. We used stratified sampling to recruit participants from our target population by email. Recruitment was conducted globally via gatekeepers and healthcare colleagues. Both pre- and post-licensure healthcare workers in all healthcare fields and levels of experience were targeted during recruitment. Our inclusion criteria were English-speaking healthcare workers, trainees, and medical students working or studying in healthcare institutions with access to a computer, tablet, or smartphone device as well as an internet connection. Our exclusion criteria were participants who failed to complete the post-simulation survey.

Participants and data collection

Upon agreeing to participate, respondents were sent another message containing instructions and links to the online pre-simulation survey, online microsimulation training module, and the online post-simulation survey. Informed consent was obtained from respondents before they completed the surveys, all of which were stored in an encrypted, secure website. All data were kept anonymous and confidential and were only accessed by our research team members.

The simulation module

**Figure 1:**
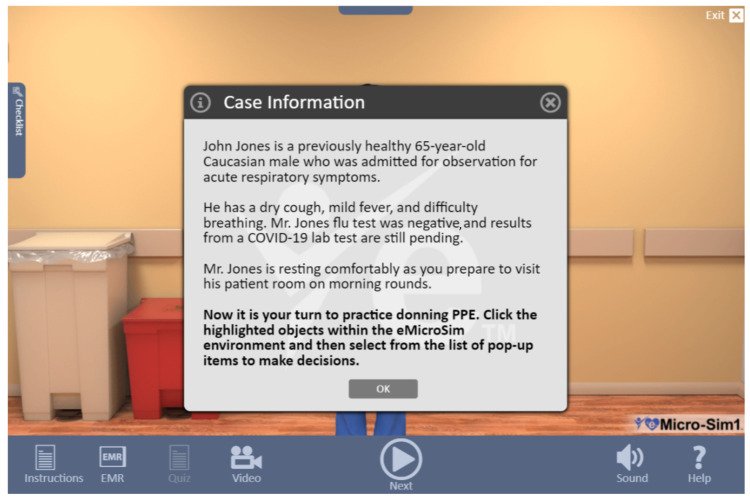
Case information Source: eTrainETC [22]. PPE: Personal protective equipment.

The PPE microsimulation virtual training module used in this study was provided by eTrain ETC, Florida, USA, and was the first iteration of the module, which was developed in partnership with Eastern Virginia Medical School, Norfolk, Virginia, to help healthcare providers reduce the risk for SARS-CoV-2 transmission [22]. The module content is based on the U.S. Centers for Disease Control (CDC) recommendations for donning and doffing of PPE. The training module was delivered entirely online, and training was undertaken by the participants as an individual via an internet connection to the eTrain website module from their remote location. No repetitions were permitted. No modifications were made to the module, and we were not aware of any limitations. No external stimuli (background noise) were used. The same patient case scenario was presented to all learners (see Figure 1). The four learning objectives of the simulation presented in the module included the following: (1) Review the basic knowledge of PPE (video demonstration provided), (2) perform PPE in a simulated environment (microsimulation virtual module), (3) assess knowledge of PPE donning and doffing (short quiz), and (4) assess critical PPE decision-making skills in a simulated case (short quiz).

**Figure 2:**
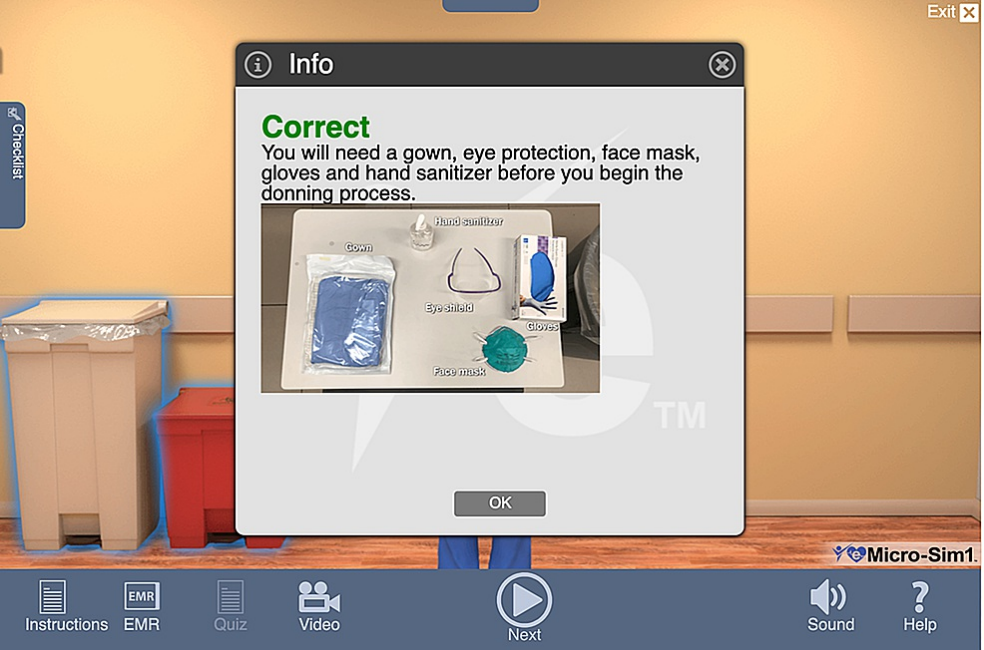
eMicro-Sim feedback Source: eTrainETC [22].

Feedback was delivered by the eTrain interface based on the decisions and actions of the participant (see Figure 2). A final report providing the quiz score and debriefing assessment was automatically generated for the participants after the training. Video recording of the training was not done.

Pilot study

To ensure content validity, the pre- and post-simulation surveys used for data collection were evaluated by three experts. Two were physicians working as medical and research professors, and one was a senior simulation educator and researcher. Based on their recommendations, questions related to age and sex were refined, and minor changes in wording were made to other questions for better clarity. Subsequently, we conducted a small-scale, preliminary pilot study (n = 12) for one week to determine the effectiveness of the components of the study, such as the clarity of the survey questions, the navigation between different components of the study, the timing allotted for each of the components of the study, and the usability of the microsimulation training module. We determined the main study to be feasible if the retention rate of the pilot study exceeded 90% and if there were no challenges faced regarding the module or the timing estimated for completion of all components. All 12 pilot study participants completed the surveys and the microsimulation virtual training without any difficulties and within the 15-minute estimated timeframe. Feedback from the pilot study participants was conveyed by an informal email to the primary investigator. No formal analysis of the pilot study was done. Feedback from pilot participants did not necessitate any changes as the issues raised were all related to suggested improvements to the platform, which was beyond our control. The participants in the pilot study were not entered into the full-scale study to avoid bias.

**Figure 3:**
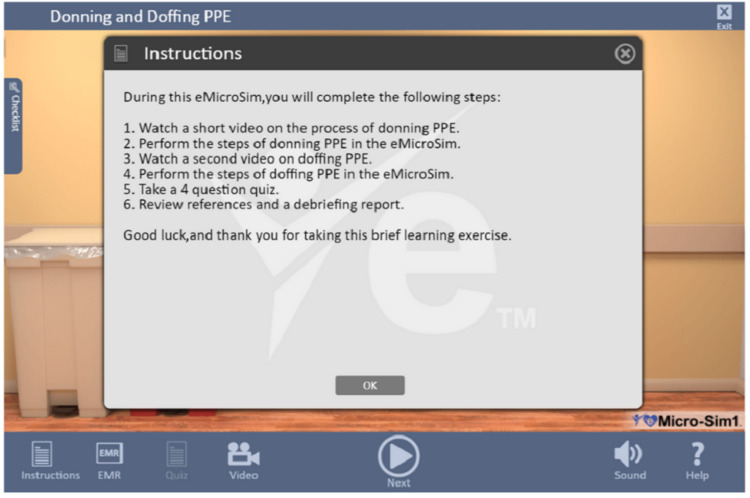
Simulation module instructions Source: eTrainETC [22]. PPE: Personal protective equipment.

The simulation training was conducted entirely online by individual participants in their setting using the eTrain module by accessing it through their own devices. No external stimuli or adjuncts were used. Participants were oriented to the simulation training module and the environment via the eMicro-Sim™ Interface Introduction Video, which explained how to use the navigation to undertake the training and complete the tasks. Before the start of the module, participants were shown a list of tasks that they would undertake to explain the steps of the training (see Figure 3) and were provided a video that demonstrated proper donning and doffing of PPE. At the start of the module, they were provided with the case information and reminders of functions to use as they participate; after that, they were prompted to start the training. A “Help” button in the menu of the module provided participants with instructions, EMR, and video, which included reminders about the training steps, cases, videos previously viewed for instructions to use the module, and the PPE donning and doffing. Thus, the Help button provided callouts of all functionalities of the module.

Data analysis

A quantitative methodology was employed for this study to investigate the perceived effectiveness of online simulation training in the proper donning and doffing of PPE. Descriptive statistics were used to analyze demographic data, which is shown in Table 1. Other items were analyzed using a paired sample t-test in Statistical Package for Social Sciences (SPSS) to compare the means of pre-training and post-training survey results. The data were checked and confirmed to meet the four assumptions that underpin the paired t-test, which are as follows: (1) The dependent variable is continuous. (2) Independent variables are matched pairs. (3) There are no significant outliers in the differences between the groups. (4) The distributions of the differences in the dependent variable between the two groups are approximately normally distributed.

**Table 1:** Descriptive statistics of the survey sample PPE: Personal protective equipment.

Variable	Category	Study Sample (n = 81)	Percentage of N (%)
Age group	20-29 years	56	69.14
	30-39 years	13	16.05
	40-49 years	7	8.64
	50-59 years	3	3.70
	>59 years	2	2.47
Gender	Female	53	65.43
	Male	26	32.10
	Prefer not to say	2	2.47
Nationality	American	5	6.17
	Canadian	2	2.47
	Egyptian	3	3.70
	Filipino	1	1.23
	Indian	6	7.41
	Jordanian	2	2.47
	Pakistani	1	1.23
	Palestinian	2	2.47
	Saudi	48	59.26
	Syrian	8	9.88
	Taiwanese	1	1.23
	Tunisian	1	1.23
Level	Prelicensure	33	40.74
	Practicing provider	48	59.26
Occupation	Medical student	18	22.22
	Nursing student	3	3.70
	Dentistry student	1	1.23
	Medical intern	4	4.94
	Dentistry intern	5	6.17
	Resident physician/Surgeon	17	20.99
	Consultant physician/Surgeon	10	12.35
	Nurse	1	1.23
	Other	22	27.16
Previous simulation participation	Yes	62	76.54
	No	19	23.46
Previous virtual simulation participation	Yes	34	41.98
	No	47	58.02
Previous formal training in PPE	Yes	53	65.43
	No	28	34.57

## Results

We received 153 responses to the pre-simulation survey and 82 responses to the post-simulation survey. We excluded the 71 participants who did not complete the post-simulation survey. Of the 82 participants who completed both surveys, one participant was excluded due to the incompleteness of responses. Demographic data are presented in Table 1; 62 participants (76.54%) reported that they have participated previously in an in-person clinical simulation session. However, only 34 participants (41.98%) reported having participated previously in virtual simulation training; 53 participants (65.43%) reported they previously received formal training (in-person) in the use of PPE before participating in this study.

Paired T-test

We used a paired t-test (see Table 2) to compare the results of the pre-training and post-training survey (n = 81). Results showed a statistically significant difference in the confidence level of observing the best practice of donning and doffing PPE before and after the training sessions (t = -3.639, p < .05). Participants also believed that virtual simulation can be an effective educational tool for clinical skills, which was reflected by a statistically significant difference pre- and post-virtual simulation sessions (t = -2.205, p < .05). Comparisons of other survey questions showed no statistical difference that are explained as follows: (1) participants’ feeling that virtual simulation is an effective way to practice donning and doffing of PPE (t = -1.270, p > .05); (2) participants’ feeling that virtual simulation will be an effective way to assess knowledge of proper donning and doffing of PPE (t = -0.823, p > .05); (3) participants’ feeling that virtual simulation will improve the practice of donning and doffing of PPE (t = -0.000, p > .05); (4) participants’ beliefs about the change in their practice of proper donning and doffing of PPE after this virtual simulation (t = -0.313, p > .05); (5) participants’ beliefs about the value in training in PPE donning and doffing through virtual simulation before doing so in healthcare settings (t = -0.210, p > .05); (6) participants beliefs regarding a clear understanding of proper donning and doffing PPE before and after the virtual training sessions (t = -1.650, p < .05); and (7) whether or not participants would like to see more virtual simulation training available to help improve their clinical skills (t = -1.033, p > .05).

**Table 2:** Paired samples correlations

Pre-simulation Survey and Post-simulation Survey Pairs	Constructs	Correlation	Significance
Pair 1	Do you feel confident in your knowledge of observing best practices in donning and doffing (putting on and taking off) personal protective equipment (PPE)?	.271	.015
Pair 2	Do you feel this virtual simulation training will be an effective way to practice proper donning and doffing of PPE?	.346	.002
Pair 3	Do you feel this virtual simulation training will be an effective way to assess your knowledge of proper donning and doffing of PPE?	.423	.000
Pair 4	Do you feel this virtual simulation training will improve your practice of proper donning and doffing of PPE?	.399	.000
Pair 5	Do you feel this virtual simulation training will improve your practice of proper donning and doffing of PPE?	.331	.003
Pair 6	Do you believe your practice of donning and doffing PPE will change after this virtual simulation training?	.277	.012
Pair 7	Do you believe this virtual simulation training will give you a clear understanding of the proper technique and sequence of donning and doffing PPE?	.539	.000
Pair 8	Do you feel virtual simulation training can be an effective educational tool for clinical skills?	.357	.001
Pair 9	Would you like to see more virtual simulation training made available to help improve your clinical skills?	.566	.000

## Discussion

Based on our findings, participants felt that after completing the virtual training, they achieved an increased level of confidence in their ability to observe best practices of donning and doffing PPE. As the virtual module provides immediate feedback to participants when they perform a task or make an active choice, participants are made aware of gaps in their knowledge and practice of skills at the moment. For example, their selection of next steps in donning and doffing of PPE serves to reveal their knowledge of and practice in the order of performance, which is essential for proper donning and doffing. Participants also felt that virtual simulation can serve as an effective educational tool for clinical skills training. Considering the limitations of face-to-face educational activities during the pandemic, we expected to receive positive perceptions from participants; however, the success of the virtual module in demonstrating itself as an effective educational option serves to also convey its usefulness beyond the pandemic.

Despite these positive perceptions, the participants did not find the virtual simulation training to be an effective way to practice or assess their knowledge of donning and doffing of PPE. We believe this to be reflective of the need to support online training with in-person practice and assessment, similar to that found by Li et al. [23], wherein video training followed by live demonstration training was determined to be a better method overall for PPE training, despite both methods being effective alone. In their study comparing telesimulation with in-person training, Lin et al. [24] found that remote training may not be as effective as that conducted in person, but their study did show an improvement in the technical and cognitive domains for providers' readiness. Ippolito et al. compared telesimulation with standard simulation in training medical students in the management of critically ill patients, and no significant difference in evaluation scores was found among the two groups and no significant difference was found in the participants' preference for telesimulation versus standard simulation [7]. These findings are further supported by the work of McCoy et al. [25] and Mikrogianakis et al. [26], wherein they detailed the effectiveness of telesimulation for educators in the context of current evidence and its use for the future of medical skills training.

Participants did not feel the training would result in an improvement or a change in their practice. We can surmise here a few possible reasons for this. The module itself may be deficient in capturing errors that would more readily be identified through in-person training and assessment. For example, the proper removal of gloves to avoid contamination, though conveyed in the instruction video, is not clearly covered in the module. For healthcare workers who understand the importance of this step, such a shortcoming in the module may be perceived as incomplete training. Second, most of our participants (59.26%) were practicing providers with active clinical experience, and as such, the training may have served as a refresher course rather than a new exposure to knowledge and practice of PPE donning and doffing, which might be of more importance to our pre-licensure trainees, who constituted 40.74% of participants in the study. 

Participants did not regard completing the virtual simulation training before doing so in a healthcare setting to be of value and did not express confidence in its ability to offer a clear understanding of proper PPE practice. This could be due to the belief that practical training is more immersive and interactive, whereas virtual training, while effective in increasing confidence levels, can be limited in its ability to offer complete and effective training. This perception could also be attributed to the deficiencies in the module. Though the module was created using evidence-based guidelines, weaknesses in the module, such as the lack of a clear demonstration of the proper doffing of gloves to avoid contamination, could explain the perception of not having confidence in its ability to offer a clear understanding of proper PPE practice rather than an overall deficiency of virtual simulation as a training method.

Most participants did not express a desire to see more clinical skills virtual simulation training made available. We assume this opinion is based on their satisfaction with their existing training opportunities or some perceived deficiencies of the training. Over 69% of our participants were in the age range of 20-29 years, a population we would expect to be digitally advanced. However, only 41.98% of our participants reported having previously participated in a virtual simulation. This lack of exposure to virtual simulation training may have biased the other 57.02% of participants toward not believing that screen/online/distance simulation could be very effective. The fact that they felt virtual simulation can serve as an effective educational tool for clinical skills training supports the value of virtual simulation training overall, and as technology advances and virtual simulation offerings improve, we expect that more and more healthcare educators, practitioners, and trainees will gravitate toward such online training options, particularly in that customization of virtual simulation modules based on trainee needs is now a prominent focus of many simulation training software companies. However, we should keep in mind the potential barriers that can exist, including having stable internet access, sufficient bandwidth, and a laptop or desktop computer, all significant considerations for low-resource communities. As well, limited technical knowledge may be a limiting factor for some participants, frustrating their efforts in their use of the training module.

In recent years, primarily because of the pandemic but also due to the rapid advancement of technology and its application to healthcare simulation, virtual training courses are becoming more and more prominent and popular. They are customizable, convenient, and not limited by space, time, or manpower. With the end of the pandemic insight and lessons gained from the social and educational restrictions experienced worldwide, the stage is being set for improvement and creative use of such courses to help increase training opportunities, assess and update skills, reduce costs, and improve patients' safety and outcomes. Also, we believe combining this training module with onsite training can serve to help learners by revealing their knowledge gaps, which can better help them prepare for in-person training and practice under observation. Such a hybrid approach can provide quality-based training that is complemented with in-person educator-delivered training with debriefing feedback tailored to the learner to deliver optimal education and help ensure learners meet competency standards.

We acknowledge that our study has limitations. Regarding research methods, there will be uncontrollable factors that can influence the findings. This study relied on volunteers, so there was a risk of selection bias as those who agreed to participate may have been different than those who chose not to participate. Although the same online module was used in the study, individuals experienced some confusion and uncertainty as they navigated the module and its functions, despite an introductory video offered for viewing which explained how to use the interface and complete the module. As the video was optional to view, participants who did not view it may have had different experiences than those who did, which may have influenced their perceptions of the training. It is also possible that participants may have had varied experiences with the module that affected their perceptions.

We were compelled to limit this study to English-speaking participants due to the microsimulation training module not being available in any other language. Although we initially limited our recruitment to participants in Riyadh, Saudi Arabia, to target a convenience sample, we made the decision to extend participation to any geographic location to diversify our participant pool. This decision was supported by the accessible nature of the virtual simulation module from anywhere in the world. However, as this decision was made later in the recruitment stage, our participants in the Middle East were considerably more than those in other countries. We had a greater number of participants who were at the prelicensure level compared to those who were practicing providers. Therefore, our findings may not be generalizable to all levels of experience.

In the surveys, we used a five-point Likert scale including the option of “neutral,” which can have varied meanings for different participants. As researchers, we cannot control peoples’ differences in personalities and how they respond to certain experiences. Learners may come to the virtual training with expectations that are not met due to their own personal understandings and interpretations. This can lead to post-survey differences that are not directly caused by the virtual training but rather influenced by previous, personal experiences.

Potential barriers for educators and learners may be the lack of stable internet access, sufficient bandwidth, and a laptop or desktop computer, which are significant considerations for low-resource communities. Also, limited technical knowledge may serve as a barrier for some participants, frustrating their efforts in their use of the training module. Accepting and navigating technology are not possible for all participants, making this an inherent barrier in its learning and application whenever we use technology [27].

To improve this work, future researchers should use more volunteers who are captured using diverse methods of sampling to minimize the risk of selection bias. Extending the study to a broader geographic location will also diversify the participant pool further, leading to more accurate results. Also, a randomized controlled trial of virtual simulation training and in-person simulation training may help to compare their efficacy. Another area of research that would be of need is to offer the training, have participants demonstrate their knowledge and skill in person, and use fluorescent powder to determine any contamination that occurs.

## Conclusions

This study has shown that virtual simulation can increase the confidence levels of the donning and doffing knowledge of healthcare workers and can be an effective method for teaching clinical skills. It can serve as a precept to clinical training and practice prior to onsite training or as a refresher course to reinforce previous training. Strengths of this virtual training were the ease of access and absence of time restrictions to undertake the training, making it a suitable module for institutions with staff constraints. As well, the module is free of charge, thus making it accessible for low-resource institutions. Virtual simulation can be an effective approach to training in proper PPE donning and doffing, especially in the presence of restrictive circumstances as that posed by the COVID-19 pandemic.

## Appendices

Pre- and Post-simulation Surveys

Pre-simulation Survey Questions

Have you ever participated in clinical skills simulation training?

Have you ever participated in clinical skills virtual simulation training?

Have you received formal training in the use of PPE?

In what year were you born?

What is your gender?

What is your nationality?

What is your occupation?

Pre-simulation Survey Questions

The following questions ask about your expectations of how the virtual simulation may benefit your learning. Please rate your agreement with the following statements (Strongly Disagree, Disagree, Neutral, Agree, Strongly Agree).

1. Do you feel confident in your knowledge of observing best practices in donning and doffing (putting on and taking off) personal protective equipment (PPE)?

2. Do you feel this virtual simulation training was an effective way to practice proper donning and doffing of PPE?

3. Do you feel this virtual simulation training was an effective way to assess your knowledge of proper donning and doffing of PPE?

4. Do you feel this virtual simulation training will improve your practice of proper donning and doffing of PPE?

5. Do you believe your practice of donning and doffing PPE will change after this virtual simulation training?

6. Do you feel this virtual simulation training gave you a clear understanding of the proper technique and sequence of donning and doffing PPE?

7. Do you see value in training in PPE donning and doffing sequences and techniques through virtual simulation before doing so in a healthcare setting?

8. After participating in this simulation, do you feel virtual simulation training can be an effective education tool for clinical skills?

9. Would you like to see more virtual simulation training made available to help improve your clinical skills?

Post-simulation Survey Questions

Please rate your agreement with the following statements (Strongly Disagree, Disagree, Neutral, Agree, Strongly Agree).

1. After taking the virtual simulation training, do you feel confident in your knowledge of observing best practices in donning and doffing (putting on and taking off) personal protective equipment (PPE)?

2. Do you feel this virtual simulation training was an effective way to practice proper donning and doffing of PPE?

3. Do you feel this virtual simulation training was an effective way to assess your knowledge of proper donning and doffing of PPE?

4. Do you feel this virtual simulation training will improve your practice of proper donning and doffing of PPE?

5. Do you believe your practice of donning and doffing PPE will change after this virtual simulation training?

6. Do you feel this virtual simulation training gave you a clear understanding of the proper technique and sequence of donning and doffing PPE?

7. Do you see value in training in PPE donning and doffing sequences and techniques through virtual simulation before doing so in a healthcare setting?

8. After participating in this simulation, do you feel virtual simulation training can be an effective education tool for clinical skills?

9. Would you like to see more virtual simulation training made available to help improve your clinical skills?
